# Nonaqueous Li-Mediated Nitrogen Reduction: Taking Control of Potentials

**DOI:** 10.1021/acsenergylett.2c02697

**Published:** 2023-01-18

**Authors:** Romain Tort, Olivia Westhead, Matthew Spry, Bethan J. V. Davies, Mary P. Ryan, Maria-Magdalena Titirici, Ifan E. L. Stephens

**Affiliations:** † Department of Chemical Engineering, Imperial College London, SW7 2AZLondon, U.K.; ‡ Department of Materials, Imperial College London, SW7 2AZLondon, U.K.

## Abstract

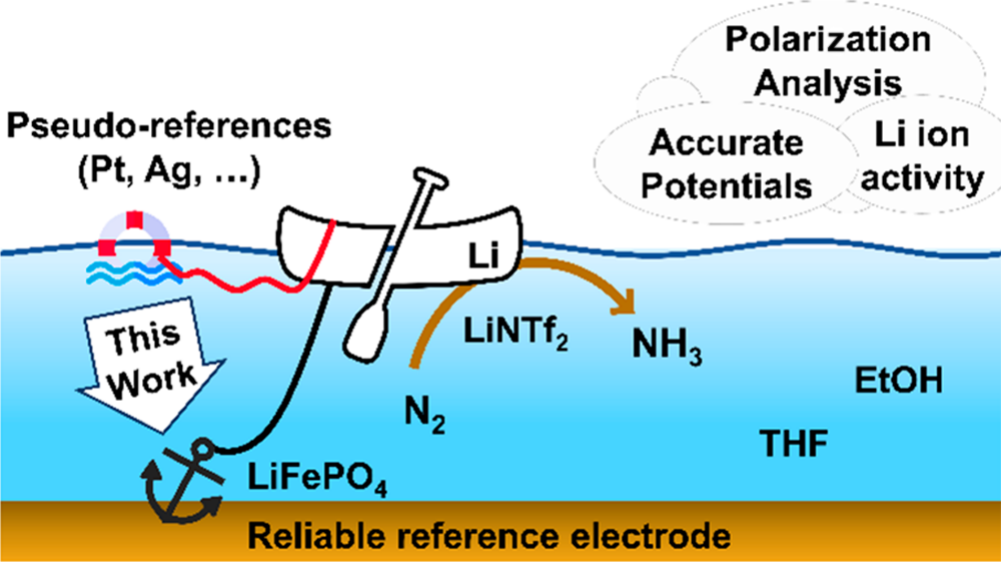

The performance of the Li-mediated ammonia synthesis has progressed dramatically since its recent reintroduction. However, fundamental understanding of this reaction is slower paced, due to the many uncontrolled variables influencing it. To address this, we developed a true nonaqueous LiFePO_4_ reference electrode, providing both a redox anchor from which to measure potentials against and estimates of sources of energy efficiency loss. We demonstrate its stable electrochemical potential in operation using different N_2_- and H_2_-saturated electrolytes. Using this reference, we uncover the relation between partial current density and potentials. While the counter electrode potential increases linearly with current, the working electrode remains stable at lithium plating, suggesting it to be the only electrochemical step involved in this process. We also use the LiFePO_4_/Li^+^ equilibrium as a tool to probe Li-ion activity changes *in situ*. We hope to drive the field toward more defined systems to allow a holistic understanding of this reaction.

Although the electrochemical nitrogen reduction reaction to ammonia is a simple transformation (eq 1), details of the mechanism remain elusive. It was only in 2019 that Andersen et al. verified that one single system–to date–is unambiguously capable of reducing N_2_ to NH_3_.^1^
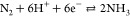
1

The lithium-mediated ammonia synthesis, initially proposed by Tsuneto et al.,^2^ allows the splitting of the N_2_ bond by direct dissociation on metallic Li; much evidence suggests that the selectivity is due to the formation of a solid electrolyte interphase over the active surface (Figure 1).^3−5^ Despite several breakthroughs in performance,^6−9^ the current understanding of this system is still largely limited, notably by the nature of experimental setups. To date, researchers in the field have mostly been using Pt or Ag wires as pseudoreferences in conventional three-electrode systems.^6−9^ However, these metals do not have a well-defined redox couple in this medium, and the equilibrium defining their redox potential is unknown.^10^ This results in a lack of independent control over the potential of each electrode, as opposed to total cell voltage, which can significantly alter the outcome of a reaction. For instance, a change in applied potential can drastically affect the selectivity of organic electrosynthetic reactions or in CO_2_ reduction.^11,12^ It also makes it difficult to establish where the losses in energy efficiency are and hence limits improvements in that regard.^13^ As with other electrochemical reactions,^10,14,15^ N_2_ reduction would benefit from an anchor to rely on toward deeper understanding of its underlying electrochemical processes: whether it is to accurately apply a desired potential to the working electrode, or to decouple cathodic from anodic contributions to total cell voltage.

**Figure 1:**
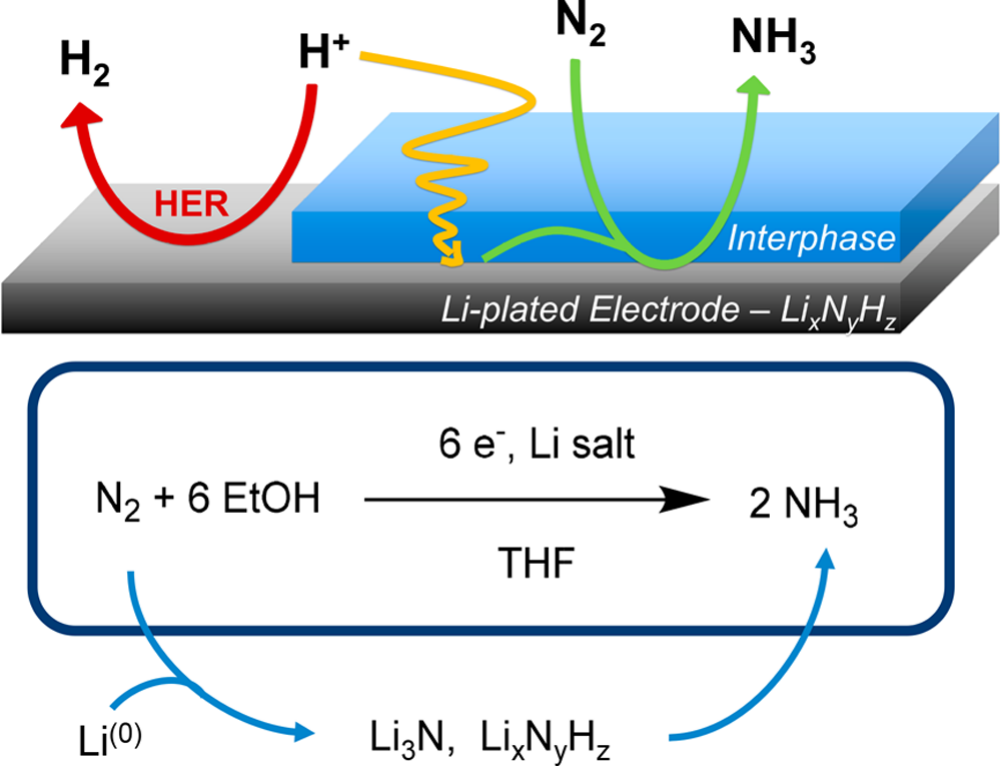
Illustration of the Li-mediated N_2_ reduction electrochemical interphase. Formation of a passivating layer from electrolyte degradation products is expected to slow down H^+^ diffusion to the active surface, using them more wisely to form NH_3_ rather than H_2_.

A *true* reference electrode ideally meets the following criteria: (i) a defined, fast, exclusive, and reversible redox equilibrium;^15^ (ii) potential reproducibility;^16^ (iii) low polarizability;^16^ and (iv) versatility for use in different electrolytes.^17,18^ To apply these criteria to N_2_ reduction, one must take note of the conventional electrolyte used. In this system, a Li salt is dissolved in THF:EtOH 99:1 v/v. It therefore makes sense to use a material that can equilibrate with Li ions to meet criterion i. As much as the research community draws inspiration from Li-ion battery science to understand and optimize this reaction,^5,7,8^ one can design a reference electrode accordingly. In this regard, battery intercalation materials such as lithium iron phosphate (LiFePO_4_) and lithium titanate (Li_4_Ti_5_O_12_) stand out. Once partially lithiated, these materials possess a phase that is in a reversible equilibrium of fast (de)intercalation of Li ions. In addition, they have a low polarizability, and their redox equilibrium potential tends to be durably stable against Li metal.^10,15^ Such reference electrodes have been successfully implemented in three-electrode batteries^19−22^ but, to the best of our knowledge, never in an electrosynthetic cell. Consequently, we will herein adapt the preparation of a LiFePO_4_ material to conditions required for the Li-mediated N_2_ reduction system and prove its superior potential stability and reproducibility—during long-time storage and electrolysis, using various electrolytes and gases—over pseudoreferences such as Pt. Finally, this reference will be used to enable deeper electrochemical analysis of the Li-mediated N_2_ reduction system via the accurate measurement of the electrode’s potentials at different operating currents.

For the reader’s attention, the work presented herein had been initially undertaken using a lithium titanate (Li_4_Ti_5_O_12_) reference electrode. However, it was observed to negatively affect the performance of the nitrogen reduction reaction, consistently delivering lower Faradaic efficiencies when present in the medium (Table S1). Since we could not identify the reason for this loss in efficiency, we decided to discard this material, in favor of LiFePO_4_.

Despite their theoretical superiority over other nonaqueous reference electrode candidates (such as Ag/Ag^+^, Li metal, Ag_2_S, etc.),^10,23,24^ intercalation materials are less popular as they require an initial conditioning step to partially lithiate/delithiate their structure to a stable phase. Previous works on controlling potentials were carried out using Ag/Ag^+^ nonaqueous references for their supposed stability, versatility, and ease of use.^25,26^ However, these electrodes suffer from several drawbacks that intercalation materials such as LiFePO_4_ can address: (i) For Ag/Ag^+^ references it is necessary to enclose the reference electrolyte within a fritted tube, creating a junction potential at the frit interface which can actually be *unstable*, *nonreproducible*, *and variable between electrolytes*,^27,28^ preventing cross-electrolyte comparison and electrochemical analysis of activity coefficients of the active species in solution^29^ (which is possible with LiFePO_4_, see discussion in Figure 4). (ii) Ag^+^ ions tend to leak through that same frit into the bulk electrolyte and can affect electrochemistry by coplating with lithium for instance.^28,30,31^ (iii) Ag/Ag^+^ references need to be freshly prepared for every experiment since the Ag salts used to make their electrolyte are light-sensitive and degrade rather quickly.^26^ (iv) These Ag salts are also hygroscopic: this accelerates degradation and may strongly affect N_2_ reduction experiments because of its sensitivity to water content in the electrolyte.^32,33^ Going back to LiFePO_4_, its reported tedious preparation—carried out using an electrolyte that is essentially the same as the one where the electrode will then be used—is not as challenging as it seems and has been swiftly adapted from commonly used Li-ion battery electrolytes (e.g., LiPF_6_ in cyclic/linear carbonate).^20,21^ In an Ar glovebox, a LiFePO_4_ disc (Ø 18 mm) was assembled in a coin cell (Figure S1a) at the positive side, against a Li metal negative electrode, separated by a glass fiber separator wetted with 1 M LiNTf_2_ (i.e., LiN(SO_2_CF_3_)_2_) in THF (omitting ethanol due to incompatibility of lithium with proton sources). Discharging the LiFePO_4_ electrode at a rate of 1.56 mA.g^–1^_LiFePO_4__ (0.01C rate) until a cutoff voltage of 4 V vs Li yielded a stable phase, reproducibly equilibrating to a potential of +3.428 ± 0.003 V vs Li (10 repeats) after relaxation, which remained stable for at least 7 days in this configuration (Figure S1b,c). After the reference electrode preparation, we went on to verify its electrochemical potential reproducibility and stability in a standard electrochemical setup for ammonia synthesis.^34^ All the following steps were undertaken in an Ar glovebox. The LiFePO_4_ disc was taken out of the coin cell and punched with an 8 mm diameter hole at its center to be assembled in a three-electrode sandwich cell (Figure S2) midway between a Mo foil working electrode and a Pt mesh counter electrode, both of 1 cm_geo_^2^ area. The 8 mm hole allows the electrolyte to flow freely between the working and counter electrodes. The as-assembled gastight cell was sequentially filled with test electrolytes containing 10 mM Ferrocene and was saturated with N_2_. For most of this work, LiNTf_2_ 1 M was chosen as a model electrolyte for its stable working electrode potential during electrolysis and for its high conductivity.^5,9^ The Ferrocene–Ferrocenium redox couple acts as an internal reference redox system,^35^ with a defined one electron redox equilibrium (Figure 2a, insert). In every test condition, a cyclic voltammogram of the electrolyte was recorded within the Ferrocene–Ferrocenium redox voltage range, at a 50 mV·s^–1^ rate. Ferrocene’s redox potential can be described by the *U*_Fc/Fc^+^_ value, defined as the average *between* the potentials at which peak cathodic (*U*_c,max_) and anodic (*U*_a,max_) currents are reached (Figure 2a), which is an estimate of its half-peak potential.^36^

**Figure 2:**
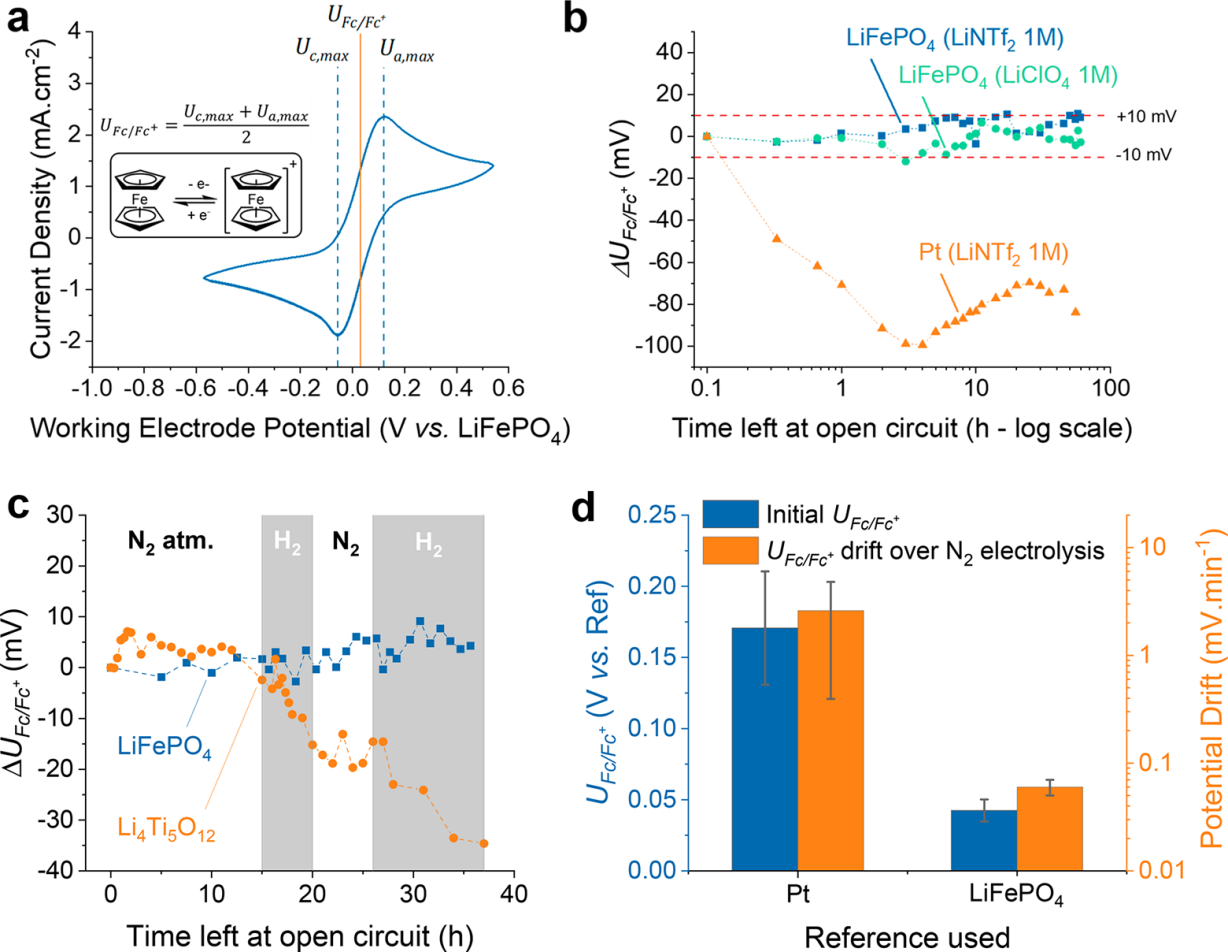
(a) Typical cyclic voltammogram at .s^−1^ of Ferrocene 10 mM with LiNTf_2_ 1 M in THF/EtOH 99:1 v/v, used to monitor changes in reference electrode potentials. Insert: scheme for the Ferrocene–Ferrocenium equilibrium. (b and c) Comparison of LiFePO_4_, Li_4_Ti_5_O_12_ and Pt electrodes stability at open circuit in 1 M LiNTf_2_ (blue squares and orange triangles, respectively) or 1 M LiClO_4_ (green circles), in THF/EtOH 99:1 v/v, saturated with (b) N_2_ or (c) H_2_ gas. (d) Reproducibility (blue) and stability (orange) comparison over the course of an electrolysis passing 10 C of charge at a constant current of −2 mA·cm_geo_^–2^ for 1 h 23 min 20 s, in LiNTf_2_ 1 M in THF/EtOH 99:1 v/v.

Figure 2b represents regular measurements of *U*_Fc/Fc^+^_ in the above-described configuration left at open circuit for 2 days. According to these measurements, LiFePO_4_ displays a stable potential for at least 60 h in different electrolytes, where slight variability (±10 mV) can be attributed to a combination of oscillation of the LiFePO_4_ potential and instrumental error. However, this oscillation is negligible when compared to the large potential drift observed when a Pt pseudoreference is used, drifting unsteadily by as much as 100 mV in less than 4 h. Assuming the largest possible drift of 10 mV (i.e., the ±10 mV oscillation observed in these measurements) over the course of the 60 h experiment depicted in Figure 2b, this counts as a drift of 0.17 mV·h^–1^, similar to values reported for Ag/Ag^+^ nonaqueous reference electrodes, reporting around 0.2 mV·h^–1^ drifts.^28,34^ It is also important to note that it is also stable in the presence of saturated H_2_ (while alternative references such as Li_4_Ti_5_O_12_ or Pt are not in such nonaqueous electrolytes, the former being too reactive (Figure 2b,c), the latter being easily poisoned^37^). This is a major feature of this reference considering the high reactivity of hydrogen and the difficulty in making a nonaqueous reference electrode in its presence, but also considering the fact that hydrogen oxidation would be the counter electrode’s reaction in an economically practical device, making this tool future-proof.^38,39^

LiFePO_4_ displays an improved electrochemical potential reproducibility across experiments, with +0.042 ± 0.008 V vs. LiFePO_4_, which is consistent with literature precedents,^18,20^ against less reproducible 0.170 ± 0.040 mV vs Pt (3 tests) (Figure 2d, blue bars). However, during electrolysis, the electrochemical environment is evolving, and such changes, like electric field or electrolyte content, can affect the potential of a reference electrode.^17,40^ The LiFePO_4_ reference stays robust in these challenging dynamic conditions (Figure 2d, orange bars). Its potential remains extremely stable over the course of a galvanostatic electrolysis at 2 mA·cm^–2^ passing 10 C of charge for 1 h 23 min 20 s, with an estimated drift of 0.06 ± 0.01 mV·min^–1^, corresponding to around 5 mV variation over the course of electrolysis, which is within the measurement error of the technique (see oscillations in Figure 2b). In comparison, the Pt pseudoreference drifts at a faster rate of 3.1 ± 1.5 mV·min^–1^.

Limited by the absence of an appropriate reference electrode for Li-mediated N_2_ reduction, current-controlled experiments have dominated the field,^1,9,25,41−43^ with only a few publications using the Ag/Ag^+^ reference electrode (which, as described earlier, may not always be optimal).^25,26^ Moving on from validating LiFePO_4_ as a reference electrode and capturing its limitations, one can use it to independently control the working electrode potential. This can strongly enhance the analytical capability of the system, providing accurate potential measurements, but also granting access to techniques that are otherwise unreliable such as potentiostatic electrolysis and electrochemical impedance spectroscopy. Figure 3 illustrates the first point. By performing electrolysis, passing 10 C of charge at constant current densities ranging between 0.1 and 10 mA·cm^–2^, accurately measuring the working and counter electrode potentials, and quantifying Faradaic efficiency to NH_3_ in each case, one can draw relations between partial current and electrode potentials.

**Figure 3:**
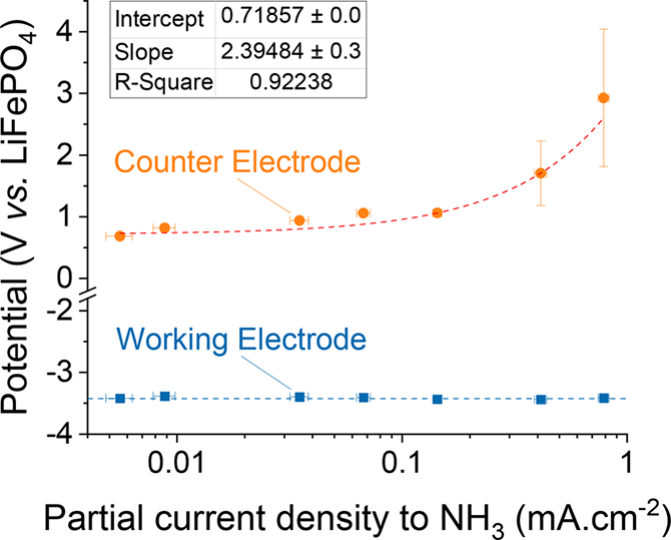
Working and counter electrode potentials recorded when passing 10 Coulomb charge at constant current densities ranging from 0.1 to 10 mA·cm_geo_^–2^, using a 1 cm^2^ Mo working electrode and Pt mesh counter electrode parallel to each other, parallel and separated by 3.6 cm with the LiFePO_4_ reference electrode midway. The produced ammonia remaining in the electrolyte post electrolysis was quantified using the Salicylate method (further details of the electrochemical cell and quantification are in the Supporting Information). Potential measurements and ohmic drop corrections are described in Figure S5. The polarization analysis displays a constant working electrode potential of −3.423 ± 0.019 V vs LiFePO_4_ and a linear increase in counter electrode potential.

Interestingly, the working electrode potential remains independent of partial current density in the tested range (0.1 to 10 mA·cm_geo_^–2^ total current) (Figure 3, blue squares), remaining at the lithium plating potential. This surprising behavior forces a re-evaluation of the classical interpretation of a typical Tafel analysis. This stable working electrode potential suggests that, beyond lithium plating, *no further electrochemical processes are limiting ammonia synthesis*, but rather physical processes, such as reagent diffusion, are limiting. More classically, the counter electrode potential rises linearly with partial current density to ammonia (Figure 3, orange circles). These measurements prove the capability of this new reference electrode to decouple anodic/cathodic electrolytic processes but also suggest that only moving away from lithium as a catalyst or limiting the energy input necessary for its deposition can help improving energy efficiency on the cathode side.

Furthermore, it is important to note that, despite the high reproducibility and stability in LiFePO_4_ equilibrium potential across different experiments, the equilibrium of a reference electrode can be affected by the surrounding environment. For instance, the equilibrium potential of Li^+^/Li on a Li metal electrode can vary by up to 500 mV depending on the solvent used^17^ or can be affected by temperature.^44^ We conjecture that such phenomena could also occur in the case of LiFePO_4_ since Li ions must first be desolvated to intercalate within the LiFePO_4_ material. By measuring *U*_Fc/Fc^+^_ in electrolytes with different concentrations of LiNTf_2_, we can effectively observe a negative deviation in measured *U*_Fc/Fc^+^_, or rather a positive deviation in LiFePO_4_ equilibrium potential with Li^+^ concentration (Figure 4), since *U*_Fc/Fc^+^_ is medium-independent.^17,27,35,45^

**Figure 4:**
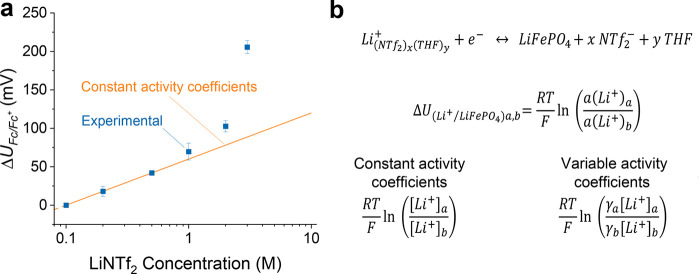
(a) Change in LiFePO_4_/Li^+^ equilibrium potential with respect to LiNTf_2_ concentration in THF/EtOH 99:1 v/v, plotted in comparison to the slope of the Nernst equation at constant activity coefficients. (b) Nernst equation for the LiFePO_4_/Li^+^ equilibrium, omitting (left) or considering (right) activity coefficient variations with concentration, the latter explaining the observed experimental deviation at higher concentrations.

As expected, at low concentrations (up to 1 M), variations in electrode equilibrium potential are captured by a simplified Nernst equation where activity coefficient variations can be neglected (Figure 4b, left). However, at higher concentrations, a deviation is observed (Figure 4a). This phenomenon can be explained by a change in the solvation sheath of Li ions with concentration, eventually affecting activity coefficients of Li ions making the LiFePO_4_/Li^+^ equilibrium potential not solely concentration-dependent (Figure 4b, right).^5,17,46^ Since the solvation environment of Li ions is so crucial to the performance and stability of Li-mediated ammonia synthesis systems,^5,9^ this tool opens avenues in the fast screening of alternative electrolytes, an opportunity that would have been missed using the Ag/Ag^+^ nonaqueous reference electrode since this requires first an ion-specific electrode^17,29^ and that cannot be achieved in the presence of a junction potential such as the one attributed to the Ag/Ag^+^ references.^27,29^ Nevertheless, we recommend that experimentalists testing different electrolytes should assess variations in LiFePO_4_ potential before making comparative conclusions.

In summary, we have demonstrated the largely improved stability and reproducibility of a lithium iron phosphate reference electrode for the nonaqueous Li-mediated N_2_ reduction system, as opposed to pseudoreferences that are standards in the field. Illustrated with the elucidation of current–potential relations, this new reference is an effective anchor for electrode potentials to be accurately measured against. It also creates opportunities for *in situ* comparison of Li-ion activity in different electrolytes, providing deeper insight into the underpinning solution chemistry, where a change in activity coefficients of Li ions can be tracked through its critical effect on the equilibrium potential of Li^+^ (de)intercalation in LiFePO_4_. Eventually, one question arises from this work: “How can we further deepen scientific insights with regard to potential control and measurement?” We suggest two paths. The first one is an engineer’s path: standardized and optimized cell geometry is essential for the acquisition of accurate, reproducible data and ideal performance in nonaqueous setups.^14,47^ For instance, reference electrode geometry and placement largely affect electrochemical impedance spectroscopy quality in a battery’s SEI characterization, and the ideal system is still a subject of debate.^22,40,48,49^ While in this work, the geometry was optimized for further studies in our laboratory, we understand that different shapes and geometries may be required. In this regard, the commercial sheets used here can be cut to any desired shape to fit a preferred cell design. In addition, this proof-of-concept can be applied to homemade coatings of LiFePO_4_ commercial powders on metal wires that may be just as flexible as metal-based pseudoreference electrodes.^19,21^ The second path is finding a way to directly correlate this measured potential to the reversible hydrogen electrode potential. Indeed, this reference potential is a standard both in aqueous and theoretical catalysis: knowing its relationship with measured potentials would enable quantification of the necessary electrochemical overpotential to drive the reaction, completing the picture given by the above analysis.^50^ In addition, H_2_ oxidation is a likely counter electrode reaction for future N_2_ reducing electrolyzers.^38,39^ Therefore, it makes sense to measure voltage against that opposite reaction to have a more precise idea of the system’s energy efficiency. Regardless, this study conveys a more solid ground as well as a wider panel of techniques for experimentation in this very active field. Moreover, we envisage that this type of reference electrode will prove extremely useful for nonaqueous organic electrosynthesis.^10,11^ While this electrode design could directly be applied to chemical reactions involving dissolved Li (e.g., Birch reduction of arenes and alkynes),^11^ we invite synthetic chemists to draw inspiration from this work and see this as a guideline for tailoring electrosynthetic reactions such as ammonia synthesis toward energy efficiency.^13^

## Supporting Information Available

Materials, experimental methods, electrochemical cell setup and measurement information, NH_3_ quantification data, table of values for measured *U*_Fc/Fc^+^_, ohmic drop correction method (PDF)
