# Supplementary material for: Nonaqueous Li-Mediated Nitrogen Reduction: Taking Control of Potentials
Source: ACS Energy Lett. 2023 Jan 18;8(2):1003–9. doi: 10.1021/acsenergylett.2c02697 (PMC9926486; doi:10.1021/acsenergylett.2c02697)
Supplement: Supplementary file 1 — nz2c02697_si_001.pdf [file nz2c02697_si_001.pdf]

Non aqueous Li-mediated nitrogen reduction: Taking control of potentials – Supplementary Information

Romain Tort,[a,b] Olivia Westhead,[b] Matthew Spry,[b] Bethan J. V. Davies,[b] Mary P. Ryan,[b] Maria-Magdalena Titirici,[a] and Ifan E. L. Stephens[b]

[a] Department of Chemical Engineering, Imperial College London, SW7 2AZ London, UK

[b] Department of Materials, Imperial College London, SW7 2AZ London, UK

Corresponding Author: Ifan E. L. Stephens – Department of Materials, Imperial College London, SW7 2AZ London, UK; Email: i.stephens@imperial.ac.uk

TABLE OF CONTENTS

1 MATERIALS AND METHODS 3

1.2. Reference electrode preparation 3
1.2.1. Coin cell assembly 3
1.2.2. Lithiation/Delithiation procedures 4

1.3. Setup for electrochemical experiments 4

1.4. Measuring the drift in potential of a studied reference electrode 4

1.5. Chronoamperometric electrolysis procedure 5

1.6. Ammonia quantification 5
1.6.1. Reagents preparation for ammonia quantification 5
1.6.2. Ammonia quantification method 6

2 FIGURES 8

Figure S 1. (a) Coin cell assembly method for $LiFePO_4$ or $Li_4Ti_5O_{12}$ conditioning, wetting the separator with 70µL $LiNTf_2$ 1M in THF, at 20°C. (b,c) Typical chronovoltammograms for galvanostatic (b) $LiFePO_4$ partial delithiation and (c) $Li_4Ti_5O_{12}$ partial lithiation at 10mA.g^{-1}$_{\text{active material}}$, showing the obtention of a stable potential of + 3.428 ± 0.003 V *vs.* Li and + 1.564 ± 0.001 V *vs.* Li, respectively. Procedure described in 1.2. 9

Figure S 2. (a) Electrochemical cell assembly diagram: 4mL electrolyte capacity, 1cm^2 working and counter electrodes surface areas, 1.7cm distance between each electrode, gas-tight with gas flow at ambient pressure (b) Assembled cell picture, (c) Cell in operation in a glovebox, closed gas line equipped with a THF bubbler to saturate inlet gas with THF. 9

Figure S 3. Typical quantification experiment as described in 1.6.2 – data analysis: (a) samples preparation volumes, (b) resulting UV-vis spectra and (c) reported maxima vs. added NH_4Cl. 10

Figure S 4. (a,b) CVs of ferrocene 10mM in 1M $LiNTf_2$ in THF/EtOH 99:1 v/v at different times of aging at open circuit, using (a) Pt and (b) $LiFePO_4$ as reference electrodes (Corresponding $UFc/Fc+$ reported in Table S 2). (c) Peak potentials difference $(Ua, max - Uc, max)$ $vs.$ time at open circuit used to calculate $UFc/Fc+$ (Figure 2b). (d) Working electrode potential recorded between ferrocene cycling steps, showing partial to full relaxation to OCP between each experiment. 10

Figure S 5. (a,b) Examples of impedance spectra of the (a) working and (b) counter electrodes for the cell assemblies made for electrolysis experiments plotted in figure 3. Recorded by potentiodynamic electrochemical impedance spectroscopy (PEIS) at open circuit voltage, with an oscillation amplitude of 20 mV at frequencies ranging from 200 kHz to 500 smHz. The ohmic drop correction was performed manually after the experiments by correcting potentials with the formula $Ueff = Urecorded - Relei$ where $Ueff$ is the corrected electrode potential, $Urecorded$ is the raw potential measured during experiments and i is the current passed during experiments. $Rele$ is the uncompensated resistance between the working (or counter) and reference electrode, collected from the PEIS measurements. $Rele$ is obtained here through fitting of the experimental data to a suitable equivalent circuit. (c) Equivalent circuit used to fit the impedance spectra and extract $Rele$ as well as other impedance contributions such as: Rct the charge transfer resistance, Cdl the double-layer capacitance, and ZW the warbug impedance describing diffusion processes. 11

3 TABLES 12

Table S 1. Comparison of Faradaic efficiency to NH_3 after a 10 C electrolysis at 2mA.cm^{-2} of a $LiNTf_2$ 1M electrolyte in THF/EtOH 99:1 v/v using Pt, $Li_4Ti_5O_{12}$ or $LiFePO_4$ as a reference electrode – Showing the interference of $Li_4Ti_5O_{12}$ in N_2 reduction experiments. 12

Table S 2. Ferrocene/Ferrocenium potential drift records for Pt, and $LiFePO_4$ across time at OCV using different salts in THF/EtOH 99:1 v/v electrolytes. 13

Table S 3. Relation between current density, working/counter electrodes potentials and faradaic efficiency to ammonia after 10C electrolysis of a $LiNTf_2$ 1M electrolyte in THF/EtOH 99:1 v/v at a corresponding current applied. 14

Table S 4. Summary of Table S 3 (mean & errors): relation between current density, electrodes potentials, Faradaic efficiency, and partial current density to NH_3. 15

1 Materials and Methods

1.1. Materials

Mo foil (99.9 %, 125 μm thick), Cu foils (99.9 %, 0.5 mm thick), Pt foil (99.95 %, 25 μm thick) and Pt mesh (99.9 %, 60 μm thick) were purchased from Goodfellow Cambridge. $LiFePO_4$ and $Li_4Ti_5O_{12}$ 18 mm diameter discs were cut out of pre-coated commercial sheets: $LiFePO_4$ coated on Al sheets (BR0188, 135 mA.h.g^{-1} capacity, 28 mg.cm^{-2} loading) purchased from MSE supplies, and $Li_4Ti_5O_{12}$ coated on Cu sheets (Nanomyte® BE-10E, 90% active material, 5% PVDF, 5% Super P 170 mA.h.g^{-1} capacity, 7.3 mg.cm^{-2} loading), purchased from NEI Corp. Lithium bis(trifluoromethanesulfonyl)imide ($LiNTf_2$, 99.9 %, < 20 ppm H_2O) was purchased from Solvionic. Ethanol (99.5 %, Extra Dry, absolute, AcroSeal™) was bought from Thermofischer™. THF (anhydrous, ≥99.9 %, inhibitor-free), Ferrocene (98 %), HCl (30 %, Suprapur®) and coin cell separators (Whatman® glass microfiber filters, Grade GF/A) were purchased from Merck KGaA Darmstadt (Sigma Aldrich). Sodium salicylate, sodium hydroxide and sodium nitroprusside were purchased from VWR. Separators were dried overnight under dynamic vacuum before use. 15 mm diameter Lithium discs, coin cell cases, spacers and wave-springs were purchased from Pi-Kem.

1.2. Reference electrode preparation

1.2.1. Coin cell assembly

Within an Ar-filled glovebox, a Li disc was mounted on stainless steel spacer and spring, placed in the negative case of a coin cell. A 18 mm diameter disc was cut out of the active materials sheets ($LiFePO_4$ or $Li_4Ti_5O_{12}$), then placed in the opposite positive case and covered with a separator, which was wetted with 70-100 µL electrolyte (1 M $LiNTf_2$ in THF). (Figure S 1a) Cell was closed with a 7-bar press.

1.2.2. Lithiation/Delithiation procedures

Partial lithiation (for $Li_4Ti_5O_{12}$) or delithiation (for $LiFePO_4$) methods were reproduced and adapted from La Mantia *et al.*[20] and Costard *et al.*[21] $LiFePO_4$/$Li_4Ti_5O_{12}$ materials assembled cells were discharged (1.56 $mA.g^{-1}_{LiFePO4}$, 0.01C rate) / charged (10 $mA.g^{-1}_{Li4Ti5O12}$, 0.06C rate) respectively, until a cut-off voltage of 4.0 or 1.3 V *vs.* Li, respectively. The cell was then left to relax to a potential plateau (+3.428 ± 0.003 V *vs.* Li for $LiFePO_4$, +1.564 ± 0.002 V *vs.* Li for $Li_4Ti_5O_{12}$), stable for days in the coin cell. (Figure S 1).

1.3. Setup for electrochemical experiments

The 3-electrode sandwich cell used in this work consists of: (i) a 1 cm^2 Mo foil working electrode, polished, dipped in 4 M HCl and sonicated in ethanol for 10 min, (ii) a Pt mesh counter electrode on a 1 cm^2 Pt foil, (iii) a reference electrode – either a Pt wire or the previously prepared $LiFePO_4$ or $Li_4Ti_5O_{12}$ disc, shaped into a ring by punching a 8 mm diameter hole at its centre –. Within an Ar-filled glovebox, the cell was assembled, with the reference midway through the two other electrodes (1.8 cm between each). Gas-tight compartments were filled with 4 mL electrolyte, and leak tested by passing Argon through. The cell was then saturated with THF-pre-saturated N_2, bubbling for 30 min at a rate of 4

mL.min^{-1} before any experiment, then turned down to 1 mL.min^{-1}. All the electrochemistry was performed in the Argon glovebox. After experiments, cell was cleaned with EtOH out of the glovebox and boiled in ultrapure water (>18.2 MΩ, Sartorius), then dried in an oven at 70°C.

1.4. Measuring the drift in potential of a studied reference electrode

To assess the extent of an undesired drift in reference electrode potential, we use the ferrocene-ferrocenium redox couple, an internal reference redox system with a defined 1 electron redox equilibrium (Figure 2a), approved as a reference for non-aqueous systems.[36] In every test condition, a voltammogram of a 1 M $LiNTf_2$ and 10 mM ferrocene in THF electrolyte was recorded, at a 50 mV.s^{-1} rate. U_{Fc/Fc^+}, the average *between* the potentials at which peak cathodic and anodic currents are reached (Figure 2a), is an estimate of its half-peak potential.[36] An important sidenote: during electrolysis, an unavoidable passivation layer forms at the working electrode (Figure 1). To avoid resistive contribution of this layer to U_{Fc/Fc^+}, post-electrolysis measurements (Figure 2d) were done after replacing the working electrode with a fresh one.

1.5. Chronoamperometric electrolysis procedure

After cell assembly, the cell was purged with N_2 for 30 min at a rate of 4 mL.min^{-1} before any experiment, then turned down to 1 mL.min^{-1}. After assessing electrolyte resistance by impedance spectroscopy (PEIS at open circuit, 20 mV oscillation, 100 kHz to 1 Hz frequency range), voltage was swept from open circuit voltage to – 4.0 V vs. $LiFePO_4$, with a cut-off current density corresponding to the final applied current during electrolysis. Once this current was reached, it was held at this level, passing a total of 10 C charge. Electrolyte resistance change was assessed post electrolysis using the same method as before, then the cell was purged

with Argon for 30 min at 4 mL.min^{-1} flow rate before disassembly. Ohmic drop was corrected manually post experiment during data processing, using PEIS data (Figure S 5).

1.6. Ammonia quantification

1.6.1. Reagents preparation for ammonia quantification

A sodium hypochlorite alkaline solution was prepared by mixing sodium hypochlorite 14 % w/w and sodium hydroxide 0.4 M in a 1:9 v/v ratio. This solution must be done shortly before every quantification experiment as the stock solution stability is limited in time.

A "salicylate – catalyst" solution was prepared by dissolving 40 g sodium salicylate powder in 50mL ultrapure water, to which 1 mL of a 50 mM aqueous sodium nitroprusside ($Na_2[Fe(CN)_5NO]\cdot 2H_2O$) solution was added. Volume was completed to 100 mL in a volumetric flask, to yield a solution containing 2.5 M sodium salicylate and 0.5 mM sodium nitroprusside catalyst. The solution was stored at 5°C in the dark for several months.

Sometimes, the sodium salicylate powder was contaminated with ammonia salts impurities. To purify it from these interferents, 40 g sodium salicylate was dissolved in 300 mL DI water, to which 50 mL of 6 M aqueous HCl was added dropwise under constant stirring. The salicylic acid precipitate was filtered and washed with three times 200 mL ultrapure water, then dried under vacuum at 40°C. For 20 g of obtained salicylic acid, the solid was dissolved in 35 mL sodium hydroxide 4 M, to which were added 580 mL of sodium nitroprusside 50 mM solution, then completed with ultrapure water to 58 mL.

1.6.2. Ammonia quantification method

Electrolyte post electrolysis was collected, with its total volume measured. 3 x 400 µL were collected alongside 400 µL of previously saved pristine electrolyte blank in separate vials. 20 µL 4 M aqueous HCl was added to each one of the four vials to trap NH_3 as NH_4Cl, which

were then placed in a 70°C water bath for 1 h to evaporate solvents. Concentrates were then redissolved in 2 mL ultrapure water.

Resulting samples were dispatched in 1 mL portions in UV-vis cuvettes, to quantify ammonia via the salicylate method: a colorimetric detection method based on the complexation of ammonia with sodium salicylate to create a blue dye. For this method, a 1 mL sample was diluted to a volume of 2 mL with ultrapure water. Then, 280 µL of the "salicylate – catalyst" solution was added, followed with 280 µL of the sodium hypochlorite alkaline solution. Samples were left to age in the dark for 45 min, then characterised by UV-vis absorption spectroscopy, measuring absorbance of light between 500 nm and 900 nm wavelengths, and measuring the difference in absorbance between the maximum (655 nm) and baseline (900 nm).

In this work, we couple this quantification experiment to the quantification method of standard addition,[33] spiking sequential amounts of a NH_4Cl standard solution (500 ppm, 9.35 mM) to the as prepared 1 mL samples and performing the salicylate quantification method to each one of these samples. By doing a linear regression on the obtained measurements, one can trace back to the ammonia present in the electrolyte, corresponding to negative of the intercept between the linear plot and the x axis (or the ratio between the slope m of the linear regression and its intercept b) (see equations $(1-3)$ and Figure S 3). $[NH_3]$ is the ammonia concentration in the electrolyte, V_{ele} is the electrolyte volume, F is the Faraday constant, C is the total charge passed during the experiment, FE is the Faradaic Efficiency to ammonia, A is the working electrode geometric surface area.

$$(1)\ [NH_3] = \frac{b}{m} \qquad (2)\ FE = \frac{3[NH_3]V_{ele}F}{C} \qquad (3)\ Yield\ Rate = \frac{[NH_3]V_{ele}}{A.Exp\ time}$$

Variances (σ_k^2) and standard errors (s_k) in measurements are estimated using the below statistical equations. A_i is the absorbance of sample with concentration c_i, $\bar{A}$ and $\bar{c}$ are the mean absorbance and concentration, respectively. m and b are the slope and intercept of the fitted linear regression. N is the overall number of measurements.

$$\sigma_A^2 = \sum_i (A_i - \bar{A})^2 - m^2 \sum_i (c_i - \bar{c})^2 \qquad \sigma_m^2 = \frac{\sigma_A^2}{\sum_i (c_i - \bar{c})^2} \qquad \sigma_b^2 = \sigma_A^2 \frac{\sum {c_i}^2}{\sum_i (c_i - \bar{c})^2}$$

$$s_k = \sqrt{\frac{\sigma_k^2}{N-1}} \; (k = A, m, b) \qquad s_{c,NH3} = \sqrt{\frac{\sigma_{c,NH3}^2}{N-1}} = C_{NH_3} \sqrt{\left(\frac{s_b}{b}\right)^2 + \left(\frac{s_m}{m}\right)^2}$$

2 Figures

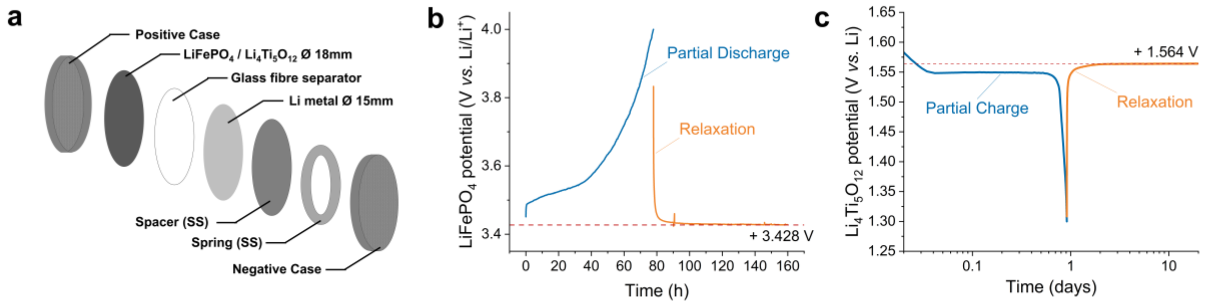

Figure S 1. (a) Coin cell assembly method for $LiFePO_4$ or $Li_4Ti_5O_{12}$ conditioning, wetting the separator with 70µL $LiNTf_2$ 1M in THF, at 20°C. (b,c) Typical chronovoltammograms for galvanostatic (b) $LiFePO_4$ partial delithiation and (c) $Li_4Ti_5O_{12}$ partial lithiation at $10mA.g^{-1}_{active\ material}$, showing the obtention of a stable potential of + 3.428 ± 0.003 V *vs.* Li and + 1.564 ± 0.001 V *vs.* Li, respectively. Procedure described in 1.2.

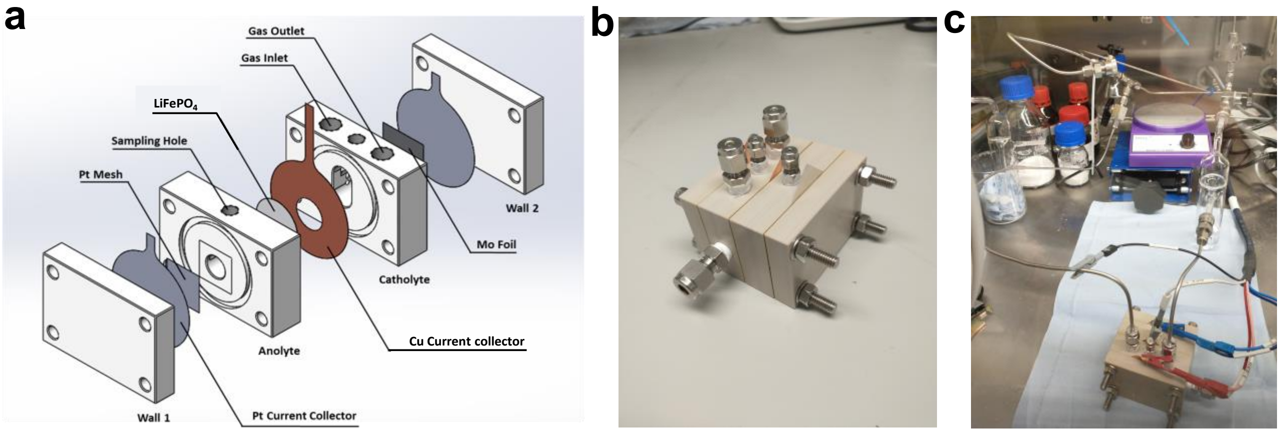

Figure S 2. (a) Electrochemical cell assembly diagram: 4mL electrolyte capacity, $1cm^2$ working and counter electrodes surface areas, 1.7cm distance between each electrode, gas-tight with gas flow at ambient pressure (b) Assembled cell picture, (c) Cell in operation in a glovebox, closed gas line equipped with a THF bubbler to saturate inlet gas with THF.

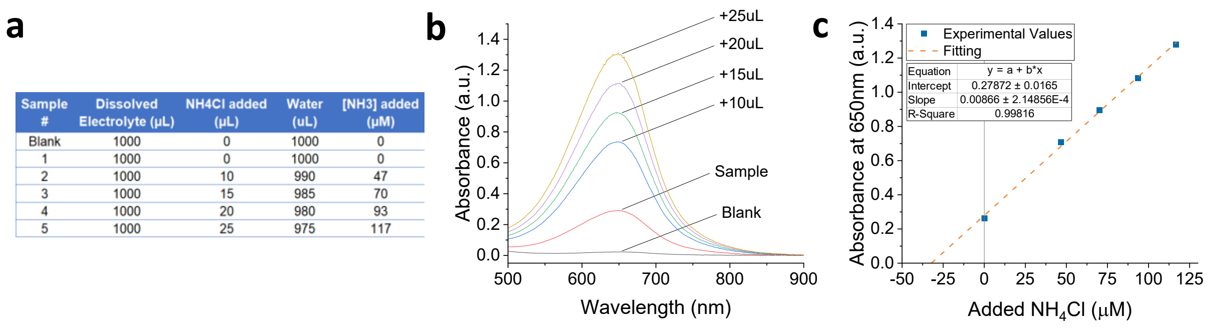

Sample #	Dissolved Electrolyte (μL)	NH4Cl added (μL)	Water (uL)	[NH3] added (μM)
Blank	1000	0	1000	0
1	1000	0	1000	0
2	1000	10	990	47
3	1000	15	985	70
4	1000	20	980	93
5	1000	25	975	117

Figure S 3. Typical quantification experiment as described in 1.6.2 – data analysis: (a) samples preparation volumes, (b) resulting UV-vis spectra and (c) reported maxima vs. added NH_4Cl.

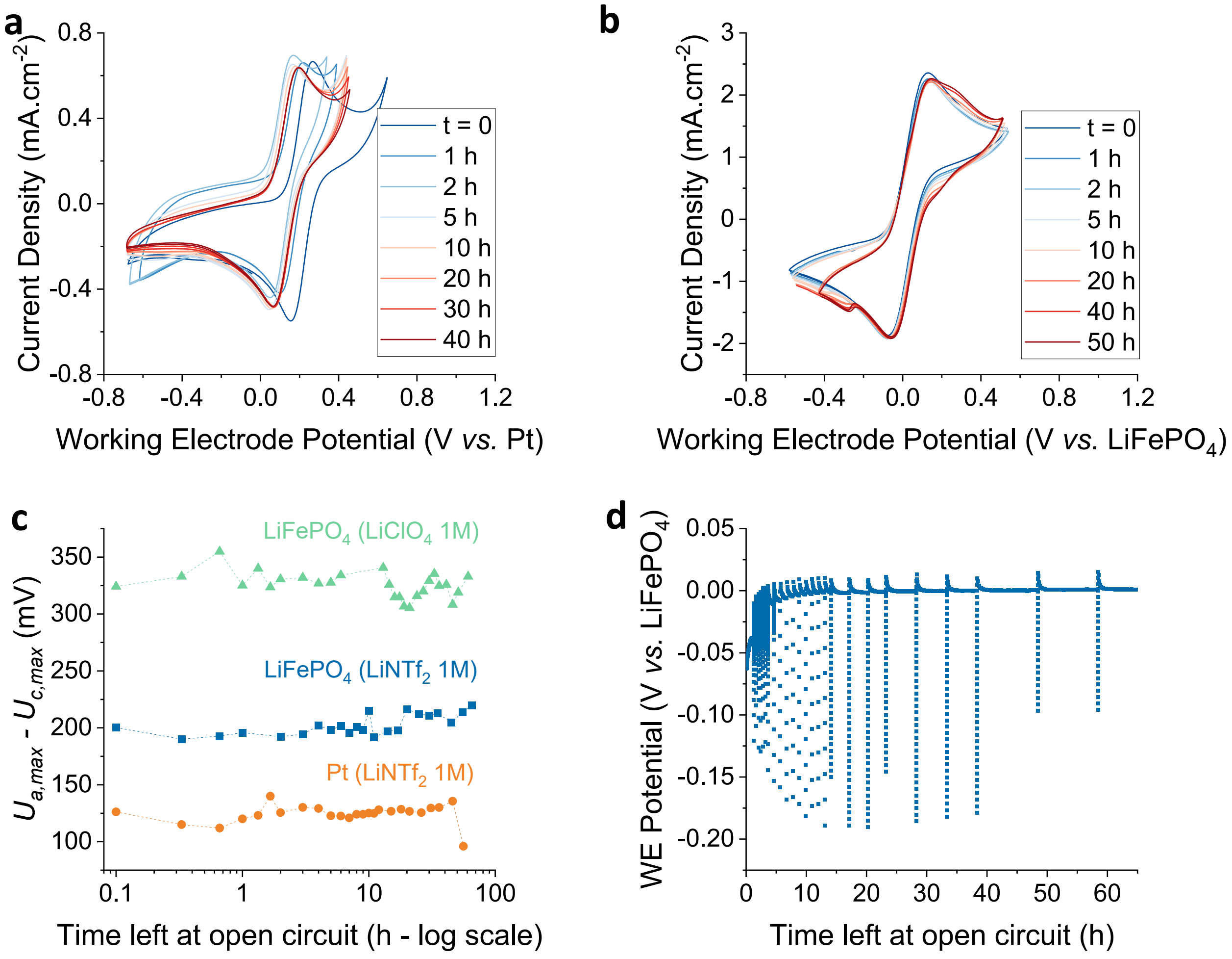

Figure S 4. (a,b) CVs of ferrocene 10mM in 1M $LiNTf_2$ in THF/EtOH 99:1 v/v at different times of aging at open circuit, using (a) Pt and (b) $LiFePO_4$ as reference electrodes (Corresponding U_{Fc/Fc^+} reported in

Table S 2). (c) Peak potentials difference ($U_{a,max} - U_{c,max}$) *vs*. time at open circuit used to calculate U_{Fc/Fc^+} (Figure 2b). (d) Working electrode potential recorded between ferrocene cycling steps, showing partial to full relaxation to OCP between each experiment.

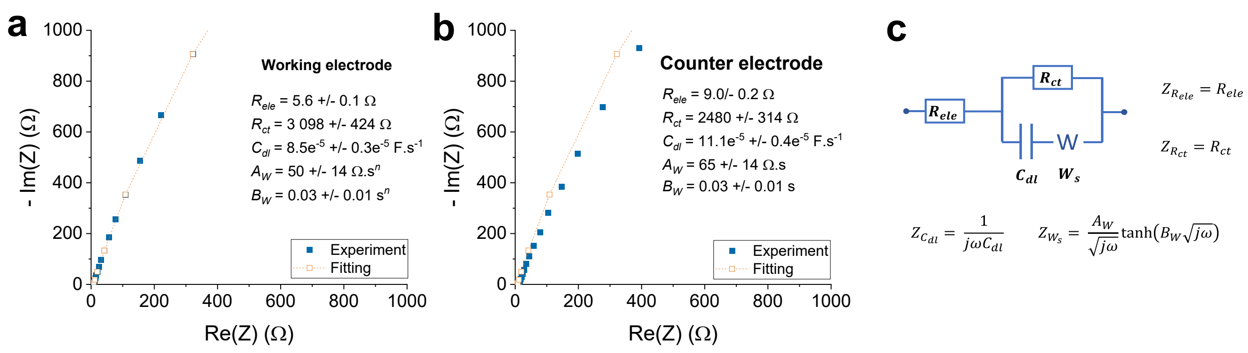

Figure S 5. (a,b) Examples of impedance spectra of the (a) working and (b) counter electrodes for the cell assemblies made for electrolysis experiments plotted in figure 3. Recorded by potentiodynamic electrochemical impedance spectroscopy (PEIS) at open circuit voltage, with an oscillation amplitude of 20 mV at frequencies ranging from 200 kHz to 500 smHz. The ohmic drop correction was performed manually after the experiments by correcting potentials with the formula $U_{eff} = U_{recorded} - R_{ele}i$ where U_{eff} is the corrected electrode potential, $U_{recorded}$ is the raw potential measured during experiments and i is the current passed during experiments. R_{ele} is the uncompensated resistance between the working (or counter) and reference electrode, collected from the PEIS measurements. R_{ele} is obtained here through fitting of the experimental data to a suitable equivalent circuit. (c) Equivalent circuit used to fit the impedance spectra and extract R_{ele} as well as other impedance contributions such as: R_{ct} the charge transfer resistance, C_{dl} the double-layer capacitance, and Z_W the warbug impedance describing diffusion processes.

3 Tables

Table S 1. Comparison of Faradaic efficiency to ammonia resulting from the electrolysis of a $LiNTf_2$ 1M electrolyte in THF/EtOH 99:1 v/v at 2mA.cm^{-2} passing 10 C of charge, using Pt, $Li_4Ti_5O_{12}$ or $LiFePO_4$ as a reference electrode – Showing the interference of $Li_4Ti_5O_{12}$ in N_2 reduction experiments.

Reference used	FE to NH_3 (%) (run 1)	FE to NH_3 (%) (run 2)	FE to NH_3 (%) (run 3)	FE to NH_3 (%) (mean)
Pt wire	7.6 ± 0.5	6.9 ± 0.2	7.8 ± 0.9	7.4 ± 0.2
$Li_4Ti_5O_{12}$	4.1 ± 0.3	4.0 ± 0.4	3.6 ± 0.5	3.9 ± 0.1
$LiFePO_4$	7.8 ± 0.2	6.7 ± 1.0	7.0 ± 0.9	7.2 ± 0.3

Table S 2. Ferrocene U_{Fc/Fc^+} drift records for Pt, and $LiFePO_4$ across time at OCV using different salts in THF/EtOH 99:1 v/v electrolytes (plotted in Figure 2b)

**Reference Electrode**	**Pt wire**	**$LiFePO_4$**	**$LiFePO_4$**
**Salt – concentration** / **Time (h)**	**$LiNTf_2$ – 1M**	**$LiNTf_2$ – 1M**	**$LiClO_4$ – 1M**
0	0	0	0
0.33	-48.9	-2.5	-2.3
0.66	-61.9	-1.7	-0.7
1	-70.9	1.5	-0.7
2	-91.6	0.2	-7.8
3	-98.9	3.5	-3.3
4	-99.5	4.2	-8.6
5	-93.3	7.3	-4.6
6	-90.2	8.7	-4.3
7	-88.4	9.1	-
8	-86.9	6.2	-
9	-83.9	7.3	-
10	-83.5	-3.6	-
11	-80.3	7.2	-
14	-77.3	9.3	0.1
17	-75.1	10.5	3.7
20	-71.2	1.4	-0.1
25	-69.7	2.2	4.2
30	-71.3	1.8	-1.2
35	-74.6	5.5	-1.4
45	-73.0	6.1	2.9
50	--	10.0	-2.6
60	-83.9	8.2	-0.3

Table S 3. Relation between current density, electrodes potentials and faradaic efficiency to NH_3 after electrolysis at constant current, passing 10 C of charge in a $LiNTf_2$ 1M electrolyte in THF/EtOH 99:1 v/v.

	$U_{working\ electrode}$ (V *vs.* $LiFePO_4$)			$U_{counter\ electrode}$ (V *vs.* $LiFePO_4$)			FE to NH_3 (%)		
Current ($mA.cm^{-2}$)	run 1	run 2	run 3	run 1	run 2	run 3	run 1	run 2	run 3
0.1	- 3.438	- 3.421	- 3.402	+ 0.676	+ 0.669	+ 0.712	4.1 ± 0.4	6.6 ± 1.0	6.0 ± 0.8
0.2	- 3.342	- 3.430	- 3.380	+ 0.936	+ 0.751	+ 0.774	1.5 ± 2.0	4.5 ± 0.2	3.5 ± 0.2
0.5	- 3.442	- 3.380	- 3.372	+ 0.921	+ 0.913	+ 0.988	8.2 ± 0.5	6.9 ± 0.8	5.9 ± 1.5
1	- 3.410	- 3.402	- 3.403	+ 1.023	+ 1.152	+ 1.005	7.5 ± 3.3	7.1 ± 0.6	6.8 ± 0.3
2	- 3.437	- 3.434	- 3.432	+ 1.057	+ 1.062	+ 1.056	7.8 ± 0.2	6.7 ± 1.0	7.0 ± 0.9
5	- 3.427	- 3.462	- 3.459	+ 1.219	+ 1.142	+ 2.748	9.3 ± 0.6	7.6 ± 1.3	6.9 ± 0.3
10	- 3.407	- 3.434	- 3.402	+ 2.069	+ 5.131	+ 1.501	8.7 ± 0.8	7.7 ± 0.5	7.2 ± 1.1

Table S 4. Summary of Table S 3 (mean & errors): relation between current density, electrodes potentials, Faradaic efficiency, and partial current density to NH_3 (plotted in Figure 3).

Current ($mA.cm^{-2}$)	$U_{working\ electrode}$ (V *vs.* $LiFePO_4$)	$U_{counter\ electrode}$ (V *vs.* $LiFePO_4$)	FE to NH_3 (%)	Partial current to NH_3 ($\mu A.cm^{-2}$)
0.1	– 3.420 ± 0.010	+ 0.686 ± 0.013	5.6 ± 0.7	5.6 ± 0.8
0.2	– 3.384 ± 0.025	+ 0.821 ± 0.059	4.4 ± 0.5	8.8 ± 0.9
0.5	– 3.398 ± 0.022	+ 0.941 ± 0.024	7.0 ± 0.7	35.0 ± 3.3
1	– 3.405 ± 0.002	+ 1.060 ± 0.046	6.7 ± 0.5	67.3 ± 4.6
2	– 3.434 ± 0.001	+ 1.061 ± 0.002	7.2 ± 0.3	143.3 ± 6.6
5	– 3.437 ± 0.011	+ 1.705 ± 0.522	8.3 ± 0.5	413.3 ± 26.2
10	– 3.414 ± 0.010	– 2.926 ± 1.115	7.9 ± 0.4	786.7 ± 44.1
